# Atherogenic Index of Plasma in Metabolic Syndrome—A Systematic Review and Meta-Analysis

**DOI:** 10.3390/medicina61040611

**Published:** 2025-03-27

**Authors:** Leia Mossane Andraschko, Gabi Gazi, Daniel-Corneliu Leucuta, Stefan-Lucian Popa, Bogdan Augustin Chis, Abdulrahman Ismaiel

**Affiliations:** 1 Faculty of Medicine, “Iuliu Hatieganu” University of Medicine and Pharmacy, 400349 Cluj-Napoca, Romania; leia.mossane@yahoo.de (L.M.A.); gabigazi99@gmail.com (G.G.); 2 Department of Medical Informatics and Biostatistics, “Iuliu Hatieganu” University of Medicine and Pharmacy, 400349 Cluj-Napoca, Romania; 3 2nd Department of Internal Medicine, “Iuliu Hatieganu” University of Medicine and Pharmacy, 400006 Cluj-Napoca, Romania; popa.stefan@umfcluj.ro (S.-L.P.); bogdan_a_chis@yahoo.com (B.A.C.)

**Keywords:** atherogenic index of plasma, biomarkers, diabetes mellitus, metabolic syndrome

## Abstract

*Background and Objectives*: Numerous studies have explored the biomarker atherogenic index of plasma (AIP) in relation to metabolic syndrome (MetS), showing its potential utility in assessing this condition. However, the existing evidence remains inconsistent and inconclusive. Therefore, this study aimed to evaluate the association between AIP and MetS and assess its predictive accuracy. *Materials and Methods:* A comprehensive search of PubMed, EMBASE, and Scopus was conducted using a predefined search strategy to identify relevant studies. Eligible studies diagnosed MetS based on the International Diabetes Federation criteria. The primary outcomes were the mean difference (MD) in AIP between MetS patients and healthy controls, as well as the area under the curve (AUC) for AIP in predicting MetS. *Results*: Thirteen studies involving 17,689 participants met the inclusion criteria and were included in the systematic review and meta-analysis. AIP levels were significantly higher in MetS patients compared to healthy controls, with an MD of 0.309 (95% CI 0.214, 0.405). In contrast, the difference in AIP levels between type 2 diabetes mellitus (T2DM) patients with MetS and normoglycemic MetS patients was not statistically significant (MD 0.142, 95% CI −0.091, 0.376). The predictive accuracy of AIP for MetS yielded an AUC of 0.864 (95% CI 0.856, 0.871). *Conclusions*: AIP levels are significantly elevated in MetS patients compared to healthy individuals, supporting AIP’s potential role as a biomarker for MetS. However, AIP levels did not differ significantly between T2DM patients with MetS and normoglycemic MetS patients. The predictive accuracy of AIP for MetS is acceptable, indicating that AIP may serve as a useful tool in MetS diagnosis. Further research is warranted to clarify its diagnostic and prognostic significance in clinical settings.

## 1. Introduction

Metabolic syndrome (MetS) is a multifaceted group of interrelated metabolic disturbances that markedly elevate the risk of cardiovascular disease (CVD) and type 2 diabetes mellitus (T2DM). Its prevalence has been rising worldwide, paralleling the increasing rates of obesity and physical inactivity. Globally, MetS affects millions of individuals with a global prevalence ranging from 12.5% to 31.4%, varying by diagnostic criteria, and is notably higher in the Eastern Mediterranean Region and the Americas [1].

MetS is defined by a cluster of metabolic abnormalities, such as central obesity, abnormal lipid levels, high blood pressure, and insulin resistance. These components synergistically contribute to the development of a pro-inflammatory and pro-thrombotic state, predisposing individuals to accelerated atherosclerosis and subsequent CVD events [2].

The atherogenic index of plasma (AIP) was first proposed by Dobiásová as a biomarker to identify plasma atherosclerosis [3]. AIP, defined as the logarithmic value of the ratio between serum triglycerides (TGs) and high-density lipoprotein cholesterol (HDL-C), acts as a valuable indicator of atherogenic dyslipidemia. This index quantitatively reflects the balance between atherogenic and anti-atherogenic lipoprotein fractions and has emerged as a valuable tool in assessing CVD risk associated with MetS. A growing body of research indicates that the AIP is a highly effective marker for predicting CVD risks [3,4,5].

In clinical practice, AIP serves as a valuable metric for assessing lipid profile atherogenicity beyond traditional lipid parameters. Elevated AIP levels indicate a predominance of atherogenic lipoproteins, such as VLDL and LDL, along with reduced protective HDL-C levels, a dyslipidemia profile characteristic of MetS and its pathophysiological association with atherosclerosis. Validating AIP as a reliable biomarker holds significant clinical implications, particularly in resource-limited settings, where cost-effective and accessible screening tools are essential for early risk stratification and targeted intervention in metabolic and cardiovascular diseases.

Despite AIP’s potential as a predictor of CVD risk in MetS, inconsistencies in prior studies hinder its precise role in risk stratification. Previous research has been limited by factors such as smaller sample sizes, lack of a specific focus on MetS, and regional variations that affect the generalizability of findings. Many studies also fail to account for variations in diagnostic criteria for MetS, further complicating the interpretation of AIP’s clinical significance. This paper, as a systematic review and meta-analysis, addresses these gaps by consolidating evidence from a range of studies, providing a comprehensive analysis of AIP’s association with MetS. By synthesizing diverse data, this study aims to clarify AIP’s clinical relevance in identifying high-risk individuals, support the development of personalized treatment strategies, and guide future research in the areas of MetS and CVD.

## 2. Materials and Methods

This systematic review and meta-analysis was conducted following the guidelines outlined in the Preferred Reporting Items for Systematic Reviews and Meta-Analyses (PRISMA) 2020 statement [6].

### 2.1. Data Sources and Strategy

We performed a comprehensive search across PubMed, Embase, and Scopus electronic databases to identify observational studies evaluating the AIP in MetS. The conducted search strategy is as follows: PubMed—((“Metabolic Syndrome” [Mesh]) OR (“Metabolic Syndrome” [All Fields])) AND ((“Atherogenic index of plasma”) OR (AIP)); EMBASE—(“Metabolic Syndrome”/exp OR “Metabolic Syndrome”) AND (“Atherogenic index of plasma” OR “AIP”); Scopus (title, abstract, keywords)—((“Metabolic Syndrome” [Mesh]) OR (“Metabolic Syndrome” [All Fields])) AND ((“Atherogenic index of plasma”) OR (AIP)). Additionally, we manually screened the reference lists of included studies to identify any potentially missed publications. The literature search was independently conducted by two investigators (L.A. and G.G.) from the inception of the databases until 3 December 2023. Discrepancies were resolved through discussion to reach a consensus. No filters or restrictions regarding publication period, country, or language were applied. Titles and abstracts were initially screened for eligibility, followed by full-text reviews of studies meeting the inclusion and exclusion criteria. Data extraction was carried out independently by two investigators (L.A. and G.G.) and verified by a third investigator (A.I.), with disagreements resolved by reviewing the original articles. The extracted data included author names, publication year, country, study design, population characteristics, sample size, AIP levels, mean age, sex distribution, BMI, MetS diagnostic criteria, AIP values expressed as mean ± SD, median (IQR), or AUC, and the primary study outcome, all of which are summarized in this manuscript.

### 2.2. Inclusion Criteria

The inclusion criteria for including original articles in our systematic review and meta-analysis were as follows: (1) observational studies, including cohort, cross-sectional, or case–control designs, evaluating AIP levels or its predictive performance in MetS as primary or secondary outcomes; (2) MetS diagnosed based on the International Diabetes Federation (IDF) or the National Cholesterol Education Program Adult Treatment Panel 3 (NCEP ATP 3) criteria; (3) human studies without restrictions on sex, race, or ethnicity; and (4) publications in English, Swedish, German, French, or Romanian.

Exclusion criteria included the following: (1) studies not reporting AIP values in both MetS and control groups; (2) editorials, letters, short surveys, commentaries, case reports, conference abstracts, review articles, pediatric studies, practice guidelines, and abstracts without accompanying full-text articles.

### 2.3. Risk of Bias Assessment in Individual Studies

The quality assessment of the included studies was independently conducted by two investigators (L.A. and G.G.) using the Newcastle–Ottawa Scale (NOS) [7], which objectively evaluates the risk of bias and internal validity. Any disagreements between the investigators were resolved through discussion. Separate evaluation forms were applied for case–control and cross-sectional studies. The studies were scored based on the number of stars awarded across three domains: selection, comparability, and outcome. The total score ranged from 0 to 10 points, with studies receiving 7 or more stars considered high quality. The quality assessment was performed for descriptive purposes only and did not influence the inclusion of studies.

### 2.4. Summary Measures and Synthesis of Results

Data analysis of the systematic review and meta-analysis was conducted using R software with the Metafor package (OpenMeta Analyst) [7,8]. The primary summary measures of AIP in MetS were the MD or AUC. Between-study heterogeneity was assessed using the χ^2^-based Q-test and I^2^ statistic. Following the guidance of the *Cochrane Handbook* for assessing and quantifying heterogeneity, we classified I^2^ values as follows: 0 to 40% as low heterogeneity, 30 to 60% as moderate, 50 to 90% as substantial, and 75 to 100% as high heterogeneity [9]. For studies reporting medians and interquartile ranges (IQRs), we converted the data into mean and SD. In studies with multiple subgroups of MetS patients or control subjects, we combined the means and standard deviations of the groups to derive a single value for the entire cohort, as recommended by the *Cochrane Handbook*, when such data were not provided. Subgroup analysis was conducted according to AIP levels in T2DM and normoglycemic patients, depending on the available values from the extracted data from included studies. For all meta-analyses, we used restricted maximum likelihood random-effects models. We presented the data from each study as the estimated MD with 95% confidence intervals (CIs), including the lower bound, upper bound, standard error, and *p*-value. Statistical significance was defined as a *p*-value < 0.05. The analyses were performed only when at least two studies reported the same outcome, with available data on mean and SD, median (IQR), or AUC.

## 3. Results

### 3.1. General Results

**Figure 1:**
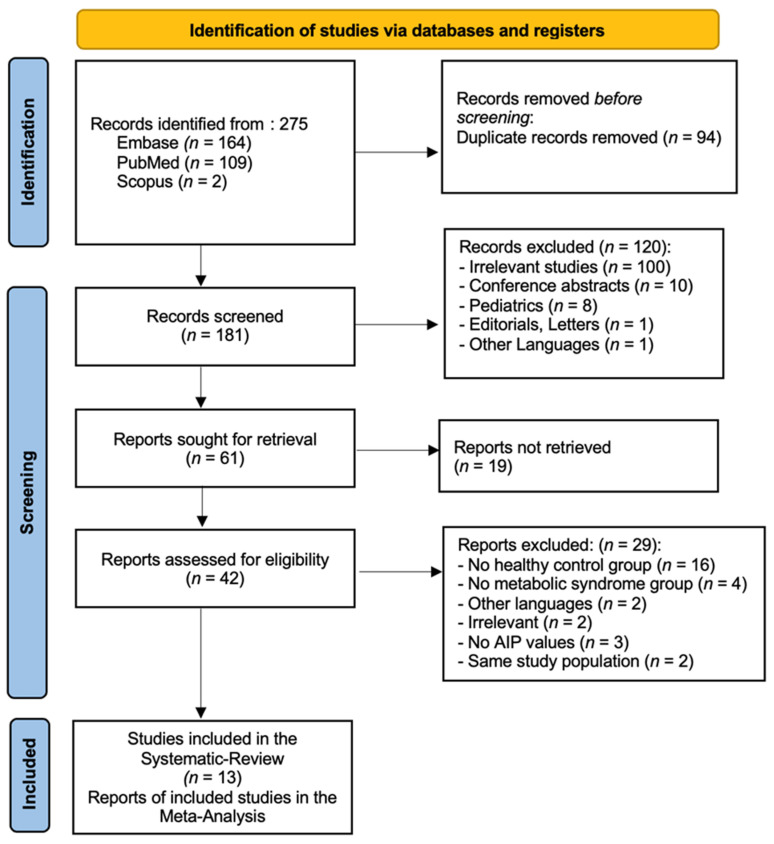
PRISMA flow diagram, illustrating the identification, screening, and inclusion stages of our systematic review and meta-analysis.

The initial search identified a total of 275 articles (PubMed = 109, EMBASE = 164, and Scopus = 2), as shown in Figure 1. Of these, 94 studies were found to be duplicates and removed. Following the removal of duplicates, 181 articles were assessed for eligibility based on the inclusion and exclusion criteria by reviewing their titles and abstracts. After the initial screening, 120 articles were excluded for the following reasons: (1) one hundred were irrelevant studies (not related to the studied topic), (2) ten were conference abstracts, (3) eight were about pediatrics, (4) one was an editorial, and (5) one was a letter to the editor. Next, we conducted a detailed review and assessment of the full texts of the remaining 42 articles to determine their eligibility. Of these, 29 articles were excluded for the following reasons: (1) sixteen had no healthy controls, (2) two were irrelevant, (3) four had no values of AIP, (4) two were in other languages, (5) two studies had the same study population, and (6) three studies had no values of AIP or participants. A total of thirteen articles were included in the qualitative synthesis, and all these studies were also incorporated into the quantitative synthesis [10,11,12,13,14,15,16,17,18,19,20,21,22].

### 3.2. Study Characteristics

A summary of the key characteristics of the included studies can be found in Supplementary Table S1. This systematic review and meta-analysis encompassed a total of 17,689 participants. According to studies that reported sex distribution, excluding one study due to non-reported sex data [21] and another study which included only females [18], the females presented a larger proportion of the included participants (females—13,944 [78.8%]; males—3575 [21.2%]). MetS was present in 4,663 subjects (26%) of the total study sample. Ten studies were conducted in Asia (Thailand *n* = 1, Taiwan *n* = 2, Iran *n* = 2, Jordan *n* = 4, Saudi Arabia *n =* 1), one in Europe (Czech Republic *n* = 1), one in Australia (Australia *n* = 1), and one in South America (Brazil *n* = 1)

### 3.3. Definition of MetS

MetS was assessed using the IDF classification in the majority [10,12,13,14,15,16,19,20,22] of studies. For four studies, MetS was assessed using the NCEP ATP 3 criteria [11,17,18,21] (*n* = 13).

### 3.4. AIP Levels in MetS

#### 3.4.1. AIP Levels in MetS Patients vs. Controls

**Figure 2:**
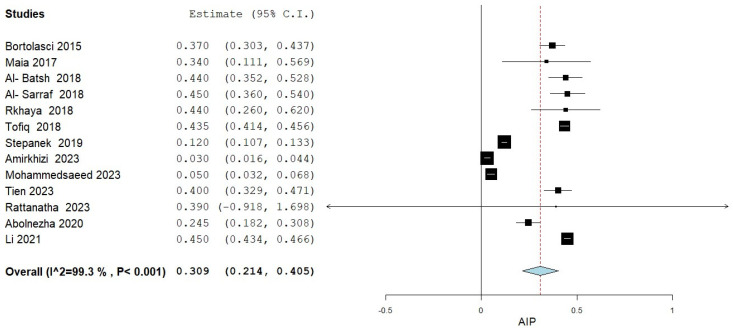
AIP levels in MetS patients vs. controls.

A total of thirteen studies assessed AIP levels, comparing MetS patients and control subjects [10,11,12,13,14,15,16,17,18,19,20,21,22]. The meta-analysis results are summarized in Figure 2. The pooled analysis comparing AIP levels in adult MetS patients to control subjects revealed an overall MD of 0.309 (95% CI: 0.214, 0.405). Significant heterogeneity was observed, with an I^2^ of 99.15% and a *p*-value < 0.001.

#### 3.4.2. Controls vs. Diabetic/Pre-Diabetic

**Figure 3:**
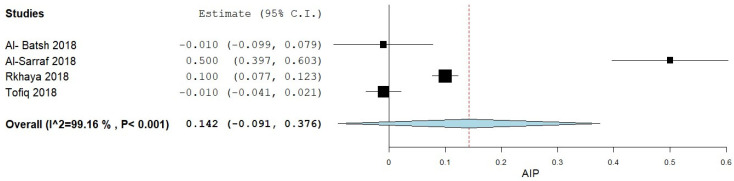
AIP levels in MetS patients according to controls vs. diabetic/pre-diabetic patients.

Additionally, a subgroup analysis was performed to compare AIP levels in controls versus diabetic/pre-diabetic patients, as shown in Figure 3. The analysis included data from four studies [12,13,14,15] which report an MD of 0.142 (95% CI −0.091, 0.376). Considerable heterogeneity was observed, with an I^2^ of 98.2% and a *p*-value < 0.001.

### 3.5. AIP in Predicting MetS

**Figure 4:**
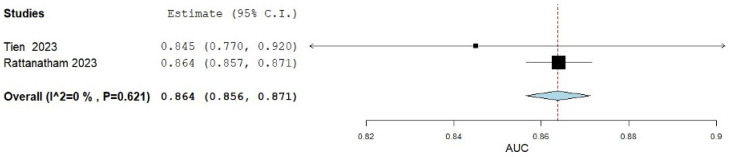
The accuracy of AIP in predicting MetS.

The predictive accuracy of AIP for identifying MetS in patients was evaluated using data from two studies. The pooled analysis results are presented in Figure 4. The analysis yielded an AUC of 0.864 (0.857, 0.871). A non-significant heterogeneity was composed of an I^2^ = 0% and a *p*-value of 0.621 [19,20].

### 3.6. Bias Evaluation

The methodological quality of the studies included in our systematic review and meta-analysis was assessed using the NOS quality assessment tool, as detailed in Supplementary Table S2. A total of thirteen articles were evaluated using this tool for cross-sectional studies [10,11,12,13,14,15,16,17,18,19,20,21,22].

Two articles received an overall score of 9/10, six articles scored 7/10, and five articles received a score of 6/10. In general, all studies had a clearly defined research objective or question. Two of the studies had a population sample that was either truly or somewhat representative of the broader target population, with a sample size that was both adequate and well justified. All studies used a validated measurement tool. Each of the thirteen studies controlled for the most significant confounding variables, as well as at least one additional factor. All studies evaluated the outcome using medical records and applied an appropriate statistical test, with results presented clearly and comprehensively.

## 4. Discussion

Recently, various scores and biomarkers have been explored in relation to MetS, particularly to assess associated cardiovascular risks, enhance the precision of current diagnostic approaches, and discover new biomarkers. In our systematic review and meta-analysis, we examined AIP levels in MetS patients. We included thirteen studies with a total of 17,689 participants in both the quantitative and qualitative synthesis. Our findings revealed that AIP levels are significantly elevated in adult MetS patients, with a reasonable diagnostic prediction accuracy.

The AIP serves as a marker for dyslipidemia and cardiovascular risk. Patients with MetS have an estimated 2-fold higher risk of developing atherosclerotic cardiovascular diseases and a 5-fold higher risk of T2DM compared to the general population [23]. MetS is also linked to faster progression of atherosclerosis, early onset of atherosclerotic cardiovascular diseases, and an increased risk of developing T2DM at an earlier age [24,25]. In recent decades, the prevalence of obesity has increased significantly, primarily driven by sedentary lifestyles and excessive caloric consumption [26]. Due to the rapid rise in obesity rates, the prevalence of MetS has seen a significant increase over the past two decades [27].

When comparing the utility of AIP to other biomarkers, it becomes evident that AIP offers a unique and valuable perspective on lipid-related cardiovascular risks. Unlike biomarkers such as fasting blood glucose (FBG) or high-sensitivity C-reactive protein (hs-CRP), which primarily reflect metabolic or inflammatory processes, AIP provides a more direct reflection of lipid imbalances, specifically the ratio of triglycerides to high-density lipoprotein (HDL) cholesterol. While FBG is essential for assessing insulin resistance and hs-CRP is useful for measuring systemic inflammation, neither captures the intricate relationship between lipid metabolism and atherosclerotic risk as well as AIP. Additionally, traditional biomarkers like total cholesterol or LDL cholesterol do not fully account for the role of triglycerides in atherogenesis, an area where AIP excels. Therefore, AIP stands out as a more holistic marker for cardiovascular risk, especially when considering its simplicity, cost-effectiveness, and ability to predict atherosclerotic events, making it an important complement to other biomarkers in both clinical and research settings.

Traditionally, a diagnosis of metabolic syndrome requires the presence of at least three of the following metabolic abnormalities: waist circumference greater than 40 inches in men and 35 inches in women, serum triglyceride levels of 150 mg/dL or higher, low high-density lipoprotein (HDL) cholesterol (below 40 mg/dL in men and below 50 mg/dL in women), fasting glucose levels of 100 mg/dL or higher, and elevated blood pressure, defined as systolic values of 130 mm Hg or higher or diastolic values of 85 mm Hg or higher [28]. Additionally, treatment for any of these conditions, such as lipid-lowering therapy, antihypertensive medication, or glucose-lowering agents, is considered equivalent to meeting the respective diagnostic criterion. While the diagnosis of MetS relies on physical examination findings and laboratory tests, obtaining a thorough patient history is crucial for effectively screening those suspected of having the condition [29]. However, it is essential to recognize that even lean individuals can exhibit features of MetS, further complicating its underlying pathogenesis [30]. Hence, it is important to find a biomarker that assists in the diagnosis of this disease with complex pathogenesis. The IDF revised its criteria in an effort to define MetS more accurately, aiming to standardize its application across diverse clinical and research settings. This updated definition was intended to enhance the ability to predict risks associated with coronary heart disease (CHD), stroke, and T2DM in various populations [31]. As per our obtained results, AIP can assist in the diagnosis of MetS and stratify patients for associated cardiovascular risk.

Present guidelines recommend evaluating the risk of atherosclerotic cardiovascular disease (ASCVD) in all patients with metabolic syndrome to formulate an effective primary prevention strategy [32]. Multiple studies have demonstrated that AIP is closely linked to a heightened risk of heart failure, stroke, and coronary artery disease [33,34,35]. Zhu X et al. found a strong correlation between the AIP and obesity [36], therefore making AIP a more susceptible marker in helping to diagnose MetS.

Studies demonstrated that central obesity is linked to a higher risk of CVD and T2DM [37]. MetS not only increases the risk for T2DM but is very predictive for its diagnosis [38,39,40,41,42]. However, the ability of MetS to predict the incidence of T2DM varies based on the criteria used to define MetS [43]. Impaired fasting glucose (IFG) can independently predict the development of T2DM, regardless of other components of MetS [44]. Hypertension is one of the diagnostic criteria for MetS, and Lioy et al. [45] did not find a correlation between AIP and hypertension alone. Our meta-analysis revealed a significant elevation in AIP levels among patients with MetS compared to healthy controls. Several published studies have explored the mechanisms underlying the role and impact of increased AIP in MetS. Although the exact mechanism remains unclear, several explanations have been proposed. AIP combines TG and HDL-C levels, serving as a comprehensive indicator of the TG to HDL-C ratio. Additionally, it reflects lipoprotein particle size, offering a more precise assessment of the pathogenicity and specificity of dyslipidemia compared to elevated TG or reduced HDL-C levels alone [46]. MetS is known to be a pro-inflammatory and pro-coagulable state due to the accumulation of lipids. Consequently, AIP levels are anticipated to be significantly elevated in patients with MetS when compared to healthy controls.

The AUC for predicting MetS was calculated at 0.864, which demonstrates a high level of accuracy and reliability as a predictive measure. This value indicates that the AIP has strong discriminatory power and can effectively differentiate between individuals with and without MetS. Given its robust performance, AIP shows promise as a supplementary tool to the existing diagnostic criteria established by the IDF. Incorporating AIP alongside traditional markers could enhance the early detection and risk stratification of MetS, ultimately improving patient outcomes and enabling more tailored interventions. Nevertheless, it is important to acknowledge the limitations of the AUC analysis, such as the limited number of included studies evaluating the AUC of AIP in MetS, potential variability in study populations, and diagnostic criteria across studies, which may impact the generalizability of the findings. While AIP shows promise as a supplementary tool to the existing diagnostic criteria established by the IDF, these results should be interpreted cautiously.

Our findings indicated that AIP levels did not show a statistically significant difference between patients with MetS and T2DM compared to healthy controls. One possible explanation for this outcome is that the study population included individuals with varying degrees of glucose intolerance, including both T2DM and pre-diabetes, which may have influenced the observed lipid profiles and attenuated the differences in AIP levels. Pre-diabetes is a condition where blood glucose levels are elevated above normal but not high enough to be diagnosed as diabetes. It is a significant concern due to its strong association with an increased risk of developing diabetes and its related complications. This intermediary condition requires careful monitoring and management to prevent progression to diabetes [47]. In the study conducted by Zhu et al. [47], the authors came to the conclusion that AIP could be used to predict the risk for T2DM. A study carried out by Yin et al. [48] pointed out that sex was an important factor for the relationship between AIP and T2DM, which was not taken in consideration in our study due to a lack of available data, which could be a reason for the non-significance of the analysis. Research has indicated that the AIP serves as a predictive marker for diabetes [45,48,49,50,51,52].

The standardization of AIP cutoffs in MetS patients remains challenging due to several factors. First, variability in study populations, including differences in demographics, genetics, and lifestyle factors, leads to inconsistencies in AIP thresholds. Additionally, the use of different diagnostic criteria for MetS, such as IDF or NCEP-ATP III, affects lipid profiles and AIP values. Moreover, there is no universally accepted AIP cutoff for MetS diagnosis, as thresholds vary depending on study methodology and population characteristics. Heterogeneity in laboratory methods for lipid measurement further complicates standardization. Lastly, ethnic and regional differences influence lipid metabolism and cardiovascular risk, preventing the establishment of a single, globally applicable AIP threshold. These factors collectively hinder the ability to define a standardized cutoff for AIP in MetS patients.

Cost-effectiveness and feasibility are key in clinical practice. Cost-effectiveness ensures that the benefits of an intervention outweigh its costs, while feasibility assesses whether it can be realistically implemented within existing healthcare systems. Both factors are crucial for ensuring that interventions are beneficial, sustainable, and accessible to patients. AIP serves as a simple, cost-effective marker for cardiovascular risk, offering insight into lipid metabolism and the potential for cardiovascular events. It is particularly useful in assessing patients at risk for metabolic syndrome, diabetes, and other conditions linked to dyslipidemia.

Our study, while comprehensive, is not without limitations that warrant consideration. First and foremost, the diverse array of study designs and participant characteristics included in our review introduces inherent variability. The diversity of ethnic and racial backgrounds in the sample was limited, as the majority of the included studies were conducted in Asia, with only one study each from Europe, South America, and Australia. It would be beneficial to investigate these findings in other populations as well. This restriction impacts the generalizability of the study results. Another limitation of this analysis is the skewed sex distribution (78.8% female) in the included studies, which may affect the generalizability of the findings to a more balanced population. Discrepancies in sample processing could influence the reliability and comparability of AIP measurements, potentially impacting the precision of our results. Additionally, it is worth noting that our subgroup analysis was based on a limited number of studies, which may have affected the reliability of the results. Heterogeneity in our analysis likely stems from differences in study design, diagnostic criteria, and participant characteristics. Variations in how MetS was defined (IDF vs. NCEP ATP 3), diverse ethnic groups, and geographical differences may all contribute to this inconsistency. Additionally, differences in AIP measurement methods and statistical approaches could further impact the results. These factors highlight the need for standardization in future research to reduce variability and enhance the reliability of findings. In light of these limitations, cautious interpretation of our findings is essential. Although our study offers important insights into the relationship between AIP levels and MetS outcomes, further research is needed to refine the findings and strengthen the reliability and applicability of our conclusions.

Furthermore, our study has several key strengths that underscore the validity and importance of our findings. Firstly, our adherence to rigorous methodology ensures transparency and comprehensiveness in our approach, enhancing the reliability of our results. By synthesizing data from multiple sources and employing standardized methodologies, we enhance the reliability and precision of our estimates, facilitating meaningful insights into the relationship between AIP and MetS. Additionally, our study benefits from a thorough evaluation of potential sources of bias and heterogeneity, including publication bias and methodological variability. Overall, our study represents a significant contribution to the existing literature on AIP and MetS, offering valuable insights that have implications for both research and clinical practice.

## 5. Conclusions

AIP levels were notably higher in patients with MetS compared to healthy controls. However, AIP levels were not significantly different between patients with pre-diabetes/T2DM and MetS compared to those with normoglycemia and MetS. The diagnostic predictive accuracy of AIP for MetS is acceptable.

## Data Availability

The analyzed data was extracted from the cited original articles as outlined in Supplementary Table S1.

## Supplementary Materials

The following are available online at https://www.mdpi.com/article/10.3390/medicina61040611/s1, Table S1: Study characteristics of included studies evaluating atherogenic index of plasma in metabolic syndrome patients, Table S2: The Newcastle-Ottawa Scale (NOS) for assessing the quality of cross-sectional studies.
