# Supplementary material for: Atherogenic Index of Plasma in Metabolic Syndrome—A Systematic Review and Meta-Analysis
Source: Medicina (Kaunas). 2025 Mar 27;61(4):611. doi: 10.3390/medicina61040611 (PMC12028871; doi:10.3390/medicina61040611)
Supplement: Supplementary file 1 [file medicina-61-00611-s001.zip › medicina-3543028-supplementary.pdf]

Supplementary Table S1. Study characteristics of included studies evaluating atherogenic index of plasma in metabolic syndrome patients

First Author / Year / Country	Study Design	Study Characteristics	Main Findings
**Bortolasci et al. / 2015 / Australia**	Cross-sectional study	• **Total Subjects:** 314<br>• **Population:** Caucasian, African and Asian with MetS<br>• **Mean age (years):** MetS: 47.3±8,9; Controls: 45.9±8.0<br>• **Sex (males):** MetS (n= 34 male), Control (n=76)<br>• **BMI:** Control 25.7 ± 4.4, MetS 29.6 ±5.0<br>• **Atherogenic index of Plasma (pmol/L):** Mean ± SD: Controls (n=224): -0.078±0.266; MetS (n=90) 0.292±0.275<br>• **AIP – AUC: -**	MetS associated with AIP and an association between BMI and AIP (MetS predicted by AIP).
**Maia et al. / 2017/ Brazil**	Cross-sectional study	• **Total Subjects:** 96<br>• **Population:** Brazilians diagnosed with Ankylosing spondylitis receiving TNF a medication and the prevalence of MetS<br>• **Mean age (years):** MetS: 41.6± 11.4; Controls: 40.1±11.5<br>• **Sex (males):** MetS (n=53): (84.1%); Control (n= 27): (81.1%)<br>• **BMI:** MetS 29.7±4.2, Control 26.8±4.3<br>• **Atherogenic index of plasma (pmol/L): Mean ± SD:** Controls (n=46): 0.34±0.24; MetS (n=17):0.68±0.46<br>• **AIP– AUC: -**	AIP was higher in the ankylosing spondylitis and MetS group compared to control.
**AL-Batsh et al. / 2018 / Jordan**	Cross-sectional study	• **Total Subjects:** 61<br>• **Population:** Jordanian patients normoglycemic with MetS or pre-DM with MetS<br>• **Mean age (years):** Normoglycemic: 49.07±10.93; PreDM: 55.68±8.4 Controls: 44.39±11.41<br>• **Sex** (males): 27.2%<br>• **BMI:** Normoglycemic: 33.85±5.67; PreDM: 33.09±6.85; Controls: 23.19 ±1.9<br>• **Atherogenic index of plasma (pmol/L): Mean ± SD:** Controls (n=29): 0.18±0.21; Normoglycemic (n= 29): 0.63±0.24; PreDM (n=30): 0.62±0.04<br>• **AIP –AUC: -**	AIP was higher in both MetS groups compared to control (p<0.001).
**AL- Sarraf et al. /2018/ Jordan**	Cross Sectional study	• **Total Subjects:** 87<br>• **Population:** Jordanian population normoglycemic with MetS and MetS with pre-DM or DM type 2<br>• **Mean age (years):** Normoglycemic: 44.5±2.0; PreDM/DM2: 52.1±2.0; Controls: 31.1±1.7<br>• **Sex (males):** 47.2%<br>• **BMI:** Controls: 22.1±1.8; Normoglycemic: 33.5±4.3; PreDM/DM2: 33.9±5.9<br>• **Atherogenic index of plasma (pmol/L): Mea**n ± **SD:** Controls (n=28): 0.2±0.2; Normoglycemic (n=29): 0.6±0.2; PreDM/DM2 (n= 30): 0.7±0.2<br>• **AIP – AUC: -**	AIP higher in both MetS groups (p<0.001).
**Rkhaya et al./2018/ Jordan**	Cross-sectional study	• **Total Subjects:** 91<br>• **Population:** Jordanian people with newly diagnosed DM2 with MetS, pre-DM or normoglycemic with MetS<br>• **Mean age (years):** Normoglycemic MetS: 44.5±2.0; PreDM/DM2 MetS: 51.8±2.0 Controls: 31.1±1.7<br>• **Sex (males):** MetS 51,6%, Control 43,2%<br>• **Atherogenic index of plasma (pmol/L): Mean ± SD:** Controls (n=30): 0.2±0.5; Normoglycemic with MetS (n=31): 0.6±0.04; PreDM/DM2 with MetS (n=30): 0.7±0.05<br>• **AIP– AUC: -**	AIP higher in both groups compared to control (p<0.001).
**Tofiq et al./2018/ Jordan**	Cross-sectional study	• **Total Subjects:** 88<br>• **Population:** Jordanians normoglycemic with MetS, preDM and DM2 with MetS<br>• **Mean age (years):** Control: 44.39±2.05; Normoglycemic with MetS: 49.07±2.00; PreDM/DM2 with MetS: 55.68±1.51<br>• **Sex (males):** 24 (27.27%)<br>• **BMI:** Control: 23.20±0.34; Normoglycemic with MetS: 33.85±1.04; PreDM/DM2 with MetS: 33.10±1.71<br>• **Atherogenic index of plasma (pmol/L): Mean** ± SD: Control (n=30): 0.19±0.04; Normoglycemic with MetS (n=30): 0.63±0.05; PreDM/DM2 with MetS (n=29): 0.62±0.07<br>• **AIP– AUC: -**	Higher AIP in normoglycemic MetS group and pre-diabetes/DM2 in comparison to control (p<0.05).

**Stepanek et al/ 2019 /Czech Republic**	Cross-sectional study	• **Total Subjects:** 376<br>• **Population:** Patients from Czech Republic with MetS<br>• **Mean age (years):** Control: 56.26 (54.24;58.28); MetS: 54.72 (52.90;56.55)<br>• **Sex (males):** 73p<br>• **BMI:** Control: 25.31(83.66;86.59) MetS: 30.69 (30.00;31.38)<br>• **Atherogenic index of plasma (pmol/L): Mean± SD:** Control (n= 188): -0.06 (-0.10; -0.02); MetS (n=188): 0.08 (0.04;0.12)<br>• **AIP- AUC:**<br>• **Measurement:** plasma	No higher values for MetS group compared to Control group due to long term use of antihypertensive and hypolipidemic medications.
**Abolnezhadian et al/ 2020/ Iran**	Cross-sectional study	• **Total Subjects:** 159<br>• **Population:** Patients in Iran with MetS and/or obesity<br>• **AIP:**<br>• **Mean age (years):** Control/MHNW: 54.70±10.76; MUNW: 52.39±10.65; MHO: 54.92±4.93; MUO: 50.90±10.46<br>• **Sex (males):** non reported sex data<br>• **BMI:** Control/ MHNW: 27.08±1.56; MUNW: 27.22±1.95; MHO: 33.38±3.33; MUO:32.98±2.96<br>• **AIP measurement Method:** calculated as log (TG/ HDL-c)<br>• **Atherogenic index of plasma (pmol/L): Mean± SD:** Control/MHNW: (n=42) 0.35±0.17; MUNW: (n=51) 0.63±0.22; MHO: (n=24) 0.45±0.16; MUO: (n=42) 0.64±0.20<br>• **AIP- AUC: -**	AIP is higher in Metabolic unhealthy and normal weight (MUNW), Metabolic unhealthy obese (MUO) compared to Metabolic healthy normal weight (MHNW) and Metabolic healthy obese (MHO).
**Li et al/ 2021/ Taiwan**	Longitudinal study or prospective Cohort study	• **Total Subjects:** 7670<br>• **Population:** People in Taiwan with MetS<br>• **Mean age (years)**: MetS: 50.94± 15.30; Controls: 48.84±15.77<br>• **Sex (males):** Control: 48.3%; MetS: 64.6%<br>• **BMI:** MetS: 27.78±4.39; Control: 22.99±3.36<br>• **Atherogenic index of plasma (pmol/L): Mea**n ± **SD:** Controls (n=6502): 0.21±0.27; MetS (n=1168): 0.66±0.26<br>• **AIP – AUC: -**	Higher AIP in MetS was observed.
**Amirkhizi et al/2023/ Iran**	Case control study	• **Total Subjects:** 195<br>• **Population:** People from Iran with MetS<br>• **Mean age (years):** Control: 37.5±5.6; MetS: 38.0±5.5<br>• **Sex (males):** Control: 34.6%, MetS: 32,3%<br>• **BMI:** Control: 31.6±1.9; MetS: 32.0±21<br>• **Atherogenic index of plasma (pmol/L) Mean± SD:** Control (n=130): 0.58±0.08; MetS (n=65): 0.61±0.08<br>• **AIP- AUC: -**	Higher AIP in MetS group compared to control group.
**Mohammedsaeed et al/2023/ Saudi Arabia**	Cross-sectional study	• **Total Subjects:** 350<br>• **Population:** Females in Saudi Arabia<br>• **Mean age (years):** Control: 22.5±9.11; MetS: 30.5±10.12<br>• **Sex (males):** only females<br>• **BMI:** Control: 25.3±9.11; MetS: 28.5±7.56<br>• **Atherogenic index of plasma (pmol/L) Mean± SD:** Control (n=206): 0.07±0.01; MetS (n= 144): 0.12±0.11<br>• **AIP- AUC: -**	Difference in AIP between MetS and control group. Patients with MetS had a higher BMI and AIP.
**Tien et al/ 2023/ Taiwan**	Cross-sectional study	• **Total Subjects:** 350<br>• **Population:** Patients in Taiwan diagnosed with Schizophrenia and treatet with antipsychotic drugs, and have MetS<br>• **Mean age (years):** Control: 40.1±9.8; MetS: 42.2±8.6<br>• **Sex (males):** 63 men, 65 women<br>• **BMI:** Control: 25.0±4.5; MetS: 29.2±4.4<br>• **Atherogenic index of plasma (pmol/L) Mean± SD:** Control (n=77): 0.2±0.2 MetS (n=51) 0.6±0.2<br>• **AIP- AUC:** 0.845 (95 % CI 0.770, 0.920)	AIP is higher compared to the control group (p<0.001).
**Rattanatham et al/2023/ Thailand**	Cross-sectional study	• **Total Subjects:** 7.852<br>• **Population:** Thai police officers with MetS<br>• **Mean age (years):** Control: 48.31±6.51; MetS: 49.11±6.18<br>• **Sex (males):** 91.2%, 7.158<br>• **BMI:** Control: 23.89±2.80; MetS: 26.52±3.43<br>• **Atherogenic index of plasma (pmol/L) Mean± SD:** Control (n=5,431): -0.05(-0.39-0.29) MetS (n=2,421): 0.34 (0.02-0.66)	AIP was higher in MetS group compared to control group (p<0.001).

		• **AIP- AUC:** 0.864 (95% CI 0.856, 0.871)<br>• **Measurement:** plasma	
AIP, Atherogenic Index of Plasma; SD, Standard Deviation; MetS, Metabolic Syndrome; DM2/PreDM2, Diabetes Mellitus type 2/ Pre-Diabetes; BMI, Body Mass Index.			

Supplementary Table S2. The Newcastle-Ottawa Scale (NOS) for assessing the quality of cross-sectional studies

**Study**	**Selection** (Maximum 5 stars)				**Comparability** (Maximum 2 stars)	**Outcome** (Maximum 3 stars)		**Score** (Total maximum 10 stars)
	**Sample representativeness**	**Sample size**	**Non-Respondents**	**Ascertainment of the exposure (risk factor)**	**Comparability**	**Assessment of the outcome**	**Statistical test**	
Bortiolasci et al 2015	*	**	-	*	*	*	*	7
Maia et al 2017	*	*	-	*	*	**	*	7
Rkhaya et al 2018	**	*	-	**	*	**	*	9
Tofiq et al 2028	*	*	-	*	*	**	*	7
AL -Batsh et al 2018	*	*	-	*	*	*	*	6
AL- Sarraf et al 2018	*	*	-	*	*	*	*	6
Stepanek et al 2019	**	*	-	**	*	**	*	9
Abolnezhadian et al 2020	*	*	-	*	*	*	*	6
Li et al 2021	*	*	-	*	*	**	*	7
Tien et al 2023	*	**	-	*	*	*	*	7
Amirkhizi et al 2023	*	*	-	*	*	**	*	7
Rattanatham et al 2023	-	**	-	*	*	*	*	6
Mohammedsaeed et al 2023	*	*	-	*	*	*	*	6
